# Comparison of Various Intrinsic Defect Criteria to Plot Kitagawa–Takahashi Diagrams in Additively Manufactured AlSi10Mg

**DOI:** 10.3390/ma16186334

**Published:** 2023-09-21

**Authors:** Mohammed Intishar Nur, Meetkumar Soni, Mustafa Awd, Frank Walther

**Affiliations:** Chair of Materials Test Engineering (WPT), Faculty of Mechanical Engineering, TU Dortmund University, Baroper Str. 303, D-44227 Dortmund, Germany; intishar.nur@tu-dortmund.de (M.I.N.); meetkumar.soni@tu-dortmund.de (M.S.); frank.walther@tu-dortmund.de (F.W.)

**Keywords:** selective laser melting, additive manufacturing, fatigue life prediction, internal defects, Kitagawa–Takahashi, finite element simulation, stress intensity factor, Paris law, AlSi10Mg

## Abstract

Selective laser melting is a form of additive manufacturing in which a high-power density laser is used to melt and fuse metallic powders to form the final specimen. By performing fatigue and tensile tests under various loading conditions, the study sought to establish the impact of internal defects on the specimens’ fatigue life. Scanning electron microscopy and finite element simulation were conducted to determine the defect characteristics and the stress intensity factor of the specimens. Four different methods were used to determine the intrinsic defect length of the specimen, using data such as grain size, yield strength, and hardness value, among others. Kitagawa–Takahashi and El-Haddad diagrams were developed using the results. A correction factor hypothesis was established based on the deviation of measured data. Using Paris law, fatigue life was determined and compared to the experimental results later. The study aims to select one or more approaches that resemble experimental values and comprehend how internal defects and loading situations affect fatigue life. This study’s findings shed light on how internal defects affect the fatigue life of selective laser-melted AlSi10Mg specimens and can aid in improving the fatigue life prediction method of additively manufactured components, provided an appropriate intrinsic crack criterion is selected.

## 1. Introduction

The industrial sector has gone through a revolution because of additive manufacturing, also known as 3D printing. By building objects up layer by layer, additive manufacturing enables precise and complex designs. Aluminum alloys are one of the most widely used materials for additive manufacturing because of their durability, lightweightness, and corrosion resistance. For aluminum alloys, selective laser melting (SLM) is a well-known additive manufacturing technique. It involves using a high-powered laser to selectively melt and fuse metal powder, layer by layer, to create a 3D object. SLM is a suitable manufacturing technology for a variety of sectors since it provides great precision and enables the production of complicated designs. Internal defects can appear during the additive manufacturing process for several reasons, including inadequate heat input, inconsistent material flow, and inappropriate printing parameters [1]. The fatigue life of aluminum alloys produced additively can be significantly impacted by internal defects. These defects can lead to stress concentrations, which can cause premature failure under cyclic loading. Porosity, lack of fusion, and cracks are common defects that can reduce the fatigue life of aluminum alloys [2]. Cracks and porosity operate as stress raisers, increasing the likelihood of failure, while porosity and a lack of fusion can decrease strength and stiffness. It can be challenging to determine the fatigue life of metals made by additive manufacturing that have internal defects. To take into account the effects of internal defects on the fatigue life of these materials, various modeling techniques have been developed. Finite element analysis (FEA) is one method for simulating the stress distribution and crack propagation behavior in the presence of internal flaws. Another strategy is to analyze the material‘s property variability using probabilistic methods and calculate the likelihood that the material would fail under various loading conditions. Both methods can be helpful in determining the fatigue life of metals produced by additive manufacturing that have internal defects and in optimizing the production process to reduce internal defects and increase the material‘s fatigue resistance. The Kitagawa–Takahashi (KT) diagram is a helpful tool for classifying how long a material will last under a certain level of stress and defect state [3]. The link between the stress amplitude and the intrinsic crack length that leads to failure for a specific material is shown in the diagram. Manufacturers can assess the safe operating range for their product and the fatigue life of their product at a specific stress level by using the KT diagram. Crack propagation is defined by a number of laws. One of the popular methods is Paris’ law. Paris’ law is a mathematical formula used to determine how quickly cracks spread through a material when it is subjected to cyclic loading. Based on the results of experimental testing, it is frequently used to calculate the fatigue strength of materials. The model explains how the rate of crack growth and the stress intensity factor relate, and it may be used to estimate how many cycles are needed for a fracture to spread to a critical size, at which point the material will break [4].

In this research work, the main priority is determining the effect of internal defects on the fatigue life of selective laser melted (SLMed) AlSi10Mg specimen. To determine fatigue life, four methods were used. For different method, different type of data was used, such as fatigue life of defect-free specimen, grain size, yield strength, hardness value, Young’s modulus, etc. These data were determined by fatigue and tensile testing, scanning electron microscopy, and finite element simulation. From hardness value calculation, the deflection of theoretical value and experimental value of maximum fatigue stress was determined, and a hypothesis for correction factor was developed. After that, the critical stress intensity factor and critical defect diameter were determined by two different methods. Both of them were used to determine fatigue life. After fatigue life was determined, it was compared with the experimentally determined value. The goal is to choose one or more method that shows a clear resemblance with experimental value and to understand how the fatigue life changes with respect to internal defect under different loading conditions.

## 2. Materials and Methods

### 2.1. Experimental Methods

A customized SLM 250 HL system with an external laser source that can produce a maximum laser power of 1000 W was used to manufacture cylindrical tensile and fatigue samples. A relative density of more than 99.54% was ensured by using an optimized scanning approach and settings. Different laser scanning parameters were used to produce the AlSi10Mg specimens’ contour and core. For generating the specimen contour, the following parameters were used: laser power *p* = 300 W, scan velocity v = 800 mm/s, and hatch distance h = 220 µm. The scan settings for the core, however, were as follows: laser power *p* = 350 W, scan velocity v = 1200 mm/s, and hatch distance h = 190 µm. The spot size D = 83 µm and layer thickness t = 50 µm were the same for both core and contour. The two-step scanning approach starts with two contour scans and then scans the core in both directions. The scanning vectors are rotated by 90 degrees for each layer [5]. Two batches of specimens A and B were created using this method. The axis of the cylinders on every specimen throughout this research is built perpendicular to the building platform at a 90° angle. The platform was not heated further during the fusion of batch A‘s powder. The laser that was used to scan the powder layer was the only source of heat. However, the building platform was heated to 200 °C when the specimens from batch B were fused. The melting pool is heated by thermal energy that is transferred upward through the specimens and into the melt pool from the building platform. As a result, the cooling rate is slowed down, and the consequential microstructure’s thermal history is changed.

For each batch of SLM AlSi10Mg, forty specimens have been produced for tensile and fatigue testing. The tensile tests were performed using a 50 kN Instron (Norwood, MA, USA) 3369 system with a 1 mm/min stroke rate. A 10 mm gauge length extensometer was used to measure the strain. For each batch, three tensile tests were conducted while accounting for scattering. Three types of fatigue tests were conducted on Instron 8872 servo-hydraulic fatigue testing system (load increase and constant amplitude tests at 20 Hz) and Shimadzu (Kyoto, Japan) USF-2000A ultrasonic fatigue testing system (constant amplitude tests at 20 kHz). First, the load increase test is conducted, which involves a stress-controlled ramp that gradually increases the stress amplitude. In this test, a dynamic extensometer with a 10 mm gauge length and WaveMatrix 2 software measure the plastic strain amplitude. The constant amplitude stress-controlled test at 20 Hz was the second type of fatigue test used. According to the findings of the load increase test’s plastic damage reaction, particular stress levels are chosen to carry out continuous amplitude tests to compare the fatigue life of both batches under equal stress levels. An identical specimen shape was utilized for the fatigue and tensile testing at 20 Hz [5]. The third fatigue test measures fatigue strength in high-cycle and very high-cycle fatigue up to 1 × 10^8^ cycles using ultrasonic constant amplitude testing at a frequency of 20 kHz. The displacement wave generated by the piezoelectric actuator is conveyed to the specimen through the horn. The free ends of the specimen undergo the same displacements in amplitude but opposite displacements in direction during harmonic vibration. A stress peak is created precisely at the center of the specimen by this displacement state. The temperature of the specimen quickly rises as a result of ultrasonic vibration. Dry air under high pressure is used as the cooling medium. Air is directed at the center of the specimen via the nozzles. Intermittent pulsing is the second defense against the specimens being heated excessively. The test is run in interrupted intervals of pausing and pulsing (pulse-pause mode) rather than in continuous pulsing mode. Throughout the test’s pauses, the specimen is given time to cool.

Microcomputed tomography is used in this study to examine how platform heating affects the development of remnant porosity and the relationship between porosity and fatigue cracks following fatigue loading. Nikon (Minato City, Japan) XT H 160 with a 160 kV X-ray gun is the system in use. The fatigue specimen is coaxially placed on the table that faces the X-ray machine. Different components of the specimen body will absorb X-rays differently while the specimen is penetrated by the X-rays. The footprint of inhomogeneity and defects can be seen in the projected 2D grey value distribution. There are 1583 2D projections made during X-ray scanning. Volumes in three dimensions were rebuilt using Nikon Metrology’s CT agent software Inspect X. Beam hardening errors and X-ray artifacts were both reduced to a minimum during reconstruction. The Volume Graphics software’s VGStudio Max 2.2 was used to import the rebuilt volumes [5]. The non-destructive defect analysis was used to confirm post-failure defects identified by scanning electron microscopy. Using a Vicker’s hardness indenter with a 100-g force, the hardness was measured. Shimadzu HMV-G21 (Kyoto, Japan) was used for the experiment. In the *X* and *Y* directions, 200 evenly spaced indentations were made. Individual indentations were separated by 400 μm [5].

### 2.2. Finite Element Model

Four models with varying load and stress ratios were developed while keeping the other aspects constant. The modeling task was completed using a combination of Microsoft Visual Studio 2019 Community Edition, Intel 1 API, and Abaqus CAE 2020. Material properties were defined by density, Depvar, and user material criteria. The models consisted of a solid cylinder with an internal defect near the wall but not touching the surface. They were subjected to a direct cyclic step, which included three tabs: Basic, incrimination, and fatigue. The cycle time period was set to 0.2, the fixed step was chosen with an increment of 0.02, and initial Fourier terms were set to 88 with a maximum of 99. The cycle increment size ranged from 1 to 4, with a maximum of 20 cycles.

A reference point RP-1 was created on the center of the lower surface of the cylinder. RP-1 was connected to the adjacent surface using the coupling constraint. The pressure was selected as the force type for all models, and the load was applied to the surface opposite to the one containing RP-1. The force magnitude was set to −1 for completely reversed models, while for complete tension models, the load magnitude was set to 0.1. Amplitude was varied according to the model’s specific applied force. ENCASTRE boundary condition was assigned to reference point RP-1, which was coupled with the lower surface. After creating .inp file for each specific model, the simulation was performed using a specified command. Once the simulation is complete, principal stress (S11, S22, S33), von Mises stress, and normal strain (E11, E22, and E33) variables were selected as output. Then, from the Element/Nodal tab, the node with the highest von Mises stress concentration was selected. In all models, it is on the edge of the defect, from where the crack initiates.

### 2.3. Theory and Calculations

**Figure 1:**
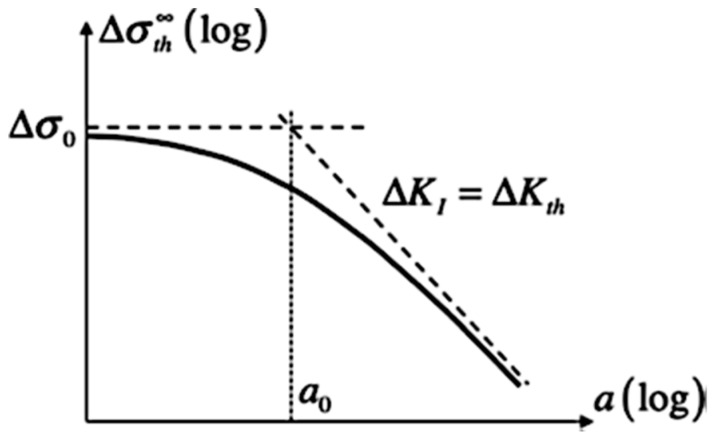
Schematic representation of the Kitagawa–Takahashi diagram.

In fracture mechanics, when predicting the stress condition (“stress intensity”) close to the crack tip or notch caused by an external load or residual stresses, the stress intensity factor K is employed [6]. It is crucial to figure out a failure standard for brittle materials. K’s value is influenced by a number of variables, including the size and position of the crack or notch, the specimen’s form, and the magnitude and distribution of material stresses. For a specimen with a natural defect, the stress intensity factor in the tip of the defect is directly related to the von Mises stress on that point and the nominal stress. It can be expressed as follows

$$K_{max}=\sigma_{Mises,\;max}/\sigma_{nominal}$$

$$K_{min}=\sigma_{Mises,\;min}/\sigma_{nominal}$$

(1) $$\Delta K=K_{max}-K_{min}$$

where $\sigma_{max}$ is the maximum von Mises at the edge of the micro defect, $\sigma_{min}$ is the minimum von Mises in the micro defect, and $\sigma_{nom}$ is the nominal stress. There are several factors that affect fatigue life, including cyclic stresses, residual stresses, material characteristics, internal flaws, grain size, temperature, design geometry, surface quality, oxidation, corrosion, etc. To determine fatigue life, instead of taking just a microscopic approach, it is typically more effective to employ a finite element approach of fatigue processes combined with fatigue and tensile tests. An effective method for integrating fracture mechanics and stress-based techniques in the fatigue design of components is the Kitagawa–Takahashi diagram [7], which is schematically depicted in Figure 1. The part is considered fail-safe when the applied stress range is lower than the defect-free material fatigue limit $\Delta \sigma_{0}$ and when the stated criteria of non-propagating crack or crack-like defect are fulfilled. At the crack size $a_{0}$, commonly referred to as the El-Haddad fictitious intrinsic crack length, the shift between these two failure modes takes place [8].

(2) $$a_{0}=\frac{1\;}{\pi }{\left(\frac{K_{th}}{Y\sigma_{0}}\right)}^{2}$$

This research developed five methods to determine the values of the threshold stress intensity factor $K_{th}$, intrinsic crack length $a_{0}$, and Kitagawa–Takahashi. The El-Haddad diagram was developed using the obtained data. After that, the critical stress intensity factor and critical defect length were also designed to determine the fatigue life of the specimen.

#### 2.3.1. Intrinsic Defect Length Calculation

The 1st method of intrinsic defect length calculation, a power law relationship of the defect size, is used to determine the fatigue strength [9]

(3) $$\sigma_{w}=\frac{K_{th}}{Y\sqrt{\pi a}}$$

Here, $\sigma_{w}$ is the fatigue limit, and Y is a dimensionless geometric factor whose value can fluctuate depending on the defect position [10]. If the defect is far from the surface, it can be approximated as 0.5. If it is on the surface, it was determined as 0.75; for an internal defect close to the surface, the factor was determined as 0.65 [11]. For load ratios smaller or equal to 0, ASTM E647-23a [12] proposes that the positive portion of the load is the sole contributor to crack propagation in metals. Therefore, in Equation (3), the expression $K_{th}=\Delta K_{th}$ is used.

To determine the intrinsic crack length $a_{0}$, the fatigue life of the defect-free specimen is also required. There is no possible way to discover the characteristics of defect-free specimens experimentally. So, using the formula below, we can roughly estimate the $\sigma_{0}$, the fatigue life of a defect-free specimen, using the hardness value we obtained from the experiment [13].

(4) $$\sigma_{0}=1.6HV\;\pm \;0.1HV$$

So, intrinsic crack length can be determined using $a_{0}=\frac{1\;}{\pi }{\left(\frac{K_{th}}{Y\sigma_{0}}\right)}^{2}$. The 2nd method uses grain size, loading ratio, and material strength to calculate $\Delta K_{th}$; a suitable formula accurately represents the significant effect of these three variables on the threshold value [14,15,16].

(5) $$\Delta K_{th}=3.28\;\sigma_{yc}\;\surd d\;\left(1-R\right)$$

Here, $\sigma_{yc}$ is the cyclic yield strength, d is the grain size, and R is the stress ratio. The morphology of the microstructure was imaged using light microscopy in the XY plane (perpendicular to the construction platform) and in the Z direction (plane perpendicular to the build platform). As the information on the grain size is achievable using microscopy, the intrinsic crack length can be determined using $a_{0}=\frac{1\;}{\pi }{\left(\frac{K_{th}}{Y\sigma_{0}}\right)}^{2}$. From various experiments, it was found that the threshold stress intensity factor has a relationship with the hardness value of the specimen. In the 3rd method, hardness value was used to determine the threshold stress intensity factor (SIF) and intrinsic crack length. $K_{th}$ increases with the increase of Vickers hardness HV. Empirically, it has been discovered that the Vickers hardness is not directly proportional to the fatigue limit of a specimen that has a notch or other defect. The difference between the threshold behavior of soft and hard materials can be stated as follows for a large range of HV [17,18,19,20], $\Delta K_{th}=C_{1}\left(HV+C_{2}\right){\left(\sqrt{area}\right)}^{\frac{1}{3}}$, where $C_{1}$ and $C_{2}$ are material-independent constants. From different experiments, it was confirmed by several researchers later that, irrespective of material properties for R = −1, threshold SIF $K_{th}$, intrinsic defect length $a_{0}$, and fatigue limit $\sigma_{w}$ for surface and internal defects can be described with these formulas [21,22,23,24,25,26,27,28,29]. For internal defect, threshold SIF, $K_{th}=2.77\times 10^{-3}\left(HV+120\right)\;a^{\frac{1}{3}}$

(6) $$\mathrm{Intrinsic}\;\mathrm{crack}\;\mathrm{length},\;a_{0}={\left(\frac{1.56\left(HV+120\right)}{1.60\;HV}\right)}^{6}$$

(7) $$\mathrm{Fatigue}\;\mathrm{limit},\;\sigma_{w}=\frac{1.56\left(HV+120\right)\;}{a^{\frac{1}{6}}}$$

In the 4th method to develop the Kitagawa diagram, the El-Haddad model was adopted. This model states that the relationship between the fatigue limit and the defect/crack size may be described as follows: $\Delta \sigma_{w}=\Delta \sigma_{o}\sqrt{\frac{a_{0}}{a+a_{0}}}$; where $\Delta \sigma_{0}$ is the defect-free material’s fatigue limit, and $\Delta \sigma_{w}$ is the material’s fatigue limit. So, intrinsic crack length

(8) $$a_{0}=\frac{a}{{\left(\frac{\sigma_{o}}{\sigma_{w}}\right)}^{2}-1}$$

For long crack propagation $a_{0}$ can be expressed as $a_{0}$ = $\frac{1}{\pi \;}{\left(\frac{K_{th,lc}}{Y\sigma_{o}}\right)}^{2}$, so

(9) $$K_{th,lc}=Y\;\sigma_{o}\sqrt{a_{0}\pi }$$

It is known as the El-Haddad parameter (described with Murakami’s area parameter). These formulations have the merit of describing the continuous transition from short cracks to long cracks, which correlates to n to infinity for $a>10\;a_{0}$ [30].

#### 2.3.2. Correction Factor Hypothesis

It can be seen that the fatigue limit can be derived from the hardness value and defect diameter. For internal defects, the formula can be described as

(10) $$\sigma_{w}=\frac{1.56\left(HV+120\right)\;}{a^{\frac{1}{6}}}$$

From the data collected from the experiment and scanning electron microscopy (SEM) for batch A specimen under 120 MPa loading, the fatigue limit is equal to 152.66 MPa according to this formula. After fatigue testing, it was found that the fatigue limit of the specimen is 95 MPa, which is significantly lower than the one found in the above formula. The error percentage is Error%=37.77%. So it can be assumed that the similar formula through which we calculated the defect-free specimen’s fatigue life provides the same error percentage. So rather than using $\sigma_{0}=\sigma_{w,0}\;\approx \;1.6HV\;\pm \;0.1HV$, the following formula can be used to determine $\sigma_{0}$

(11) $$\sigma_{0}=1.6HV-0.3777\times 1.6HV=124.46\;\mathrm{MPa}$$

Another formula was discussed to determine the fatigue limit of aluminum alloys without considering the defect diameter in this paper [31]. In this method, only the stress ratio, the ultimate tensile strength of the specimen, is considered. Here fatigue limit is [32]

(12) $$\sigma_{a}=\left(0.53-5.66\times 10^{-4}\times \sigma_{b}\right)\;\cdot \sigma_{b}$$

where $\sigma_{a}$ is the fatigue limit under R=−1, $\sigma_{b}$ is the ultimate tensile strength. For batch A, $\sigma_{b}$ = 428.94 MPa; for batch B, $\sigma_{b}$ = 450.76 MPa. So, for batch A, $\sigma_{0}\;$= $\sigma_{fatigue\;limit}$ = 123.20 MPa, and for batch B, 123.90 MPa for defect-free specimens. As can be seen here, the fatigue limit of the defect-free specimen, which was found out from this formula, is almost the same as what we found out from the correction factor hypothesis. Here, the error percentage is only 0.004%, which is almost negligible. So, it can be said that the correction factor hypothesis on the hardness value formula is better suited than the original approximation. The fatigue life of defect-free specimens found by the correction factor hypothesis determined intrinsic crack length and threshold SIF for all the methods. This correction factor hypothesis does not affect the 3rd method, as it does not use the fatigue life of defect-free specimens. 

#### 2.3.3. Critical Stress Intensity Factor Calculation

A material’s ability to resist crack extension is described by the critical stress intensity factor $K_{IC}$. Fracture strength is another name for the stress intensity factor. The determination of the material characteristic value of fracture mechanics under cyclic stress with constant amplitude is covered in ASTM E399-22 [33]. Here, two methods were used to determine critical SIF. Later, critical crack length was calculated from both of the values. In the 1st method of $K_{IC}$ calculation, it is derived from the traditional Griffith crack theory [34] to include a more precise term for strain energy release rate alongside crack surface energy γ′, crack length a, modulus E, applied stress σ, crack-tip plastic zone defect region rp, and yield strength ys, all of which can be found from load and deflection records. The square root proportionality of $E^{1/2}$ for the critical stress intensity factor $K_{c}$ has previously been used to estimate the elastic modulus E and c [6,7,35,36,37,38]. Griffith demonstrated in 1920 that a fracture would spread if the strain energy per unit of crack surface energy γ′ were greater than the material’s atomic bond energies to produce two new surfaces [35] by the formula $\sigma =\sqrt{\frac{2E\gamma \prime }{\pi a}}$, where σ is the applied stress, E is the material modulus, and a is either the entire length of a surface defect or half of its size if it is an internal crack. In this study, only internal defect is considered. In that case

(13) $$\gamma \prime =\frac{\sigma^{2}\pi a}{2E}$$

Further consideration of thermodynamics for the energy of both fracture surfaces resulted in the rate of toughness losing its definition as $G_{c}$ was redefined using a conventional toughness value as the cross-sectional area of the sample at the crack and reinserted for γ′. 

$G_{c}=2\gamma \prime$, as $G_{c}$ is the crack force per crack area beyond a critical value, $G_{c}$ actually represents the work of fracture or fracture energy of the propagating crack from maximum load to complete failure [6,7,34,36,37,38,39,40,41], so the critical stress intensity factor

(14) $$K_{Ic}=\sigma \sqrt{\pi a}\;=\sqrt{EG_{c}}$$

the stress intensity factor in mode I (tension) ($K_{I}$) reaches a critical (c) value such that fracture occurs when $K_{I}=K_{IC}$ [34,36,42] because $K_{IC}$ is derived as ${\left(EG_{c}\right)}^{1/2}$ [6,34,36,37,38,39,40,41,42]. A geometrical correction factor Y has been estimated to be 0.65 for an internal defect as a boundary condition for initial failure during flexural testing because the critical fracture toughness parameter, such as $K_{IC}$, estimates material properties reflected by crack lengths under particular loading conditions within a material [36]

(15) $$K_{Ic}=\gamma \sigma \sqrt{\pi a}=\gamma \sqrt{EG_{Ic}}=1.125\sqrt{EG_{Ic}}$$

The experimental results and equations can be rearranged as follows to determine the theoretical starter fracture length at max strength for analysis of critical defect $a_{c}$

(16) $$a_{c}=\frac{1}{\pi \;}{\left(\frac{K_{Ic}}{\sigma_{max}}\right)}^{2}$$

In the 2nd method, J-integral was used for $K_{IC}$ calculation. In light of the current rupture behavior, the determination of the deformation energy changes. The second type is present in this instance; hence, the evaluation will employ the following equations [43]

(17) $$\mathrm{The}\;J\text{-}\mathrm{integral}\;\mathrm{is}\;\mathrm{computed}\;\mathrm{using}\;J_{IC}=\frac{U}{B\left(W-a\right)}f\left(\frac{a}{w}\right)$$

Here, $B\left(W-a\right)$ is considered as the cross-sectional area for a flat specimen. For cylindrical specimens, it will be $\frac{\pi }{4}{\left(D-a\right)}^{2}$. Here, *D* = 5 mm, the function of pre-crack length and specimen width can be calculated $f\left(\frac{a}{W}\right)=2\frac{1+\alpha }{1+\alpha^{2}}$. The parameter α can be determined from the following formula:

(18) $$\alpha =\sqrt{({\left(\frac{2a}{W-a}+1\right)}^{2}+1})-\left(\frac{2a}{W-a}+1\right)$$

Here, U is deformation energy equivalent to the area underneath the Force-Displacement curve. As this formula is justified in this case, it can be said that $J_{IC}=G_{IC}$, so the critical stress intensity factor

(19) $$K_{IC}=\sqrt{E\;\frac{G_{IC}}{1-\nu^{2}}}$$

and critical crack length, $a_{c}=$ $\frac{1}{\pi \;}{\left(\frac{K_{IC}}{\sigma_{max}}\right)}^{2}$

#### 2.3.4. Fatigue Life Calculation

It is essential to learn more about the fatigue life of the part while constructing a new engineering component using a certain material. A formula that can be used to predict fatigue life can be created by combining data on fracture toughness and fatigue crack growth. It was found that integration of the fatigue crack growth rate equation, also known as Paris’ law, between the initial defect length $a_{0}$ and the critical defect length $a_{c}$, which was found at fatigue failure after the number of cycles to failure $N_{f}$, one sort of equation for determining fatigue life can be created [44]. Paris law states

(20) $$da/dN=C\Delta K^{m}$$

Here, da/dN = fatigue crack growth rate, ΔK is the stress-intensity factor range, and C, m are Paris constants that are a function of the material, environment, frequency, temperature, and stress ratio. According to theoretical considerations, it is typically impossible to estimate the parameters C and m entering Paris’ law; hence, fatigue tests must be carried out. A consistent relationship between the parameters C and m, however, was experimentally discovered by numerous authors [45,46,47]. The relationship is

(21) log C=log A+m log B

A and B are material-specific constants and depend on the stress ratio and $K_{IC}$. By comparing the empirically determined values of B with those theoretically anticipated according to the equation; an experimental evaluation is carried out to verify the validity of the suggested relationship derived based on the instability condition of the crack growth [47]. Radhakrishnan [45] gathered information about aluminum alloys from a variety of sources and presented the following least square fit relationships (with K measured in MPa and da/dN measured in m/cycle)

(22) $$log\;C=log\;\left(2.5\times 10^{-4}\right)+m\;log\;\left(4.26\times 10^{-2}\right)$$

The parameters m and $K_{IC}$ must be known in advance in order to evaluate the prediction of our suggested correlation with the experimentally determined values of B. ASM handbook [48] offers a collection of values in a figure $K_{IC}$ vs. the temperature test and the prior austenite grain size. The critical stress-intensity factor’s estimated average value is $K_{IC}=$ 35 MPa√m, with THE lowest and maximum values of 15 MPa√m and 49 MPa√m, respectively, according to handbooks [48,49,50,51]. Using the median values, it has been discovered $log\;C=log\;\left(2.5\times 10^{-4}\right)-m\;log\;\left(2.86\times 10^{-2}\right)$. The value of B changes depending on the stress ratio and fracture toughness value. In this study, for different values of $K_{IC}$ for different specimens, the values of B were changed. SIF can also be written as ΔK=Yσ√πa

(23) $$\mathrm{So},\;\Delta K^{m}=Y^{m}\sigma^{m}\pi^{m/2}a^{m/2}$$

Using $\Delta K^{m}$ in Paris’ law equation,

(24) $$da/dN=C\;\left(Y^{m}\sigma^{m}\pi^{m/2}a^{m/2}\right)$$

The defect size was integrated from the initial defect size $a_{o}$ to the final defect size at failure $a_{c}$, and the fatigue cycle limit was from 0 to the number at fatigue failure $N_{f}$ after rearranging the equation above. Thus,

(25) $$\int_{a_{0}}^{a_{c}}da=CY^{m}\sigma^{m}\pi^{m/2}a^{m/2}\;\int_{0}^{N_{f}}dN\;\mathrm{or}\;\int_{0}^{N_{f}}dN={\left(CY^{m}\sigma^{m}\pi^{m/2}\right)}^{-1}\int_{a0}^{ac}\frac{da}{a^{m/2}}\;\mathrm{So},\;\mathrm{fatigue}\;\mathrm{life},N_{f}=\frac{a_{f}^{-\left(\frac{m}{2}\right)+1}-a_{0}^{-\left(\frac{m}{2}\right)+1}}{CY^{m\;}\sigma^{m}\;\pi^{m/2}\;\left(-\frac{m}{2}+1\right)}\;\;\;\;m\neq 2.$$

This is the formula to determine Fatigue life. The assumption required for this formula is m≠2.

## 3. Results

### 3.1. Experimental Results

**Figure 2:**
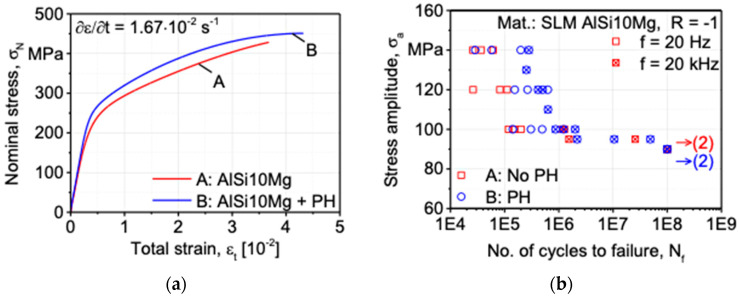
Results from tensile and fatigue test: (**a**) Stress-strain curve; (**b**) Stress-fatigue life plot [5].

The stress-strain curves for batches A and B are displayed in Figure 2a. As can be seen, batch B exhibited higher fracture strain, yield strength, and tensile strength. The curves displayed are an intermediate curve between two batches of three tensile tests. The yield strength was not considerably different statistically. Nevertheless, the average of three experiments reveals that batch B has a 30 MPa greater tensile strength. The fracture strain average is greater than batch B’s average. However, because of the non-homogeneity of the structure, batch A exhibits a higher level of property dispersion. Due to platform heating, batch B’s highly homogenous structure produces more dependable and uniform characteristics. The fatigue life of all the specimens of each batch was also determined from the fatigue test. Figure 2b shows the results of fatigue tests for batch A and batch B, respectively, with no platform heating and platform heating in the form of an S-N curve. It was performed under three sets of loading conditions of 100 MPa, 120 MPa, and 140 MPa and at two frequencies of 20 Hz and 20 kHz, respectively. At the same time, the stress ratio was kept constant at R=−1. It can be observed that the specimen with platform heating is likely to withstand a greater number of cycles until the fatigue failure than that of no platform heating involved. The positive effect of platform heating is noticed in both charts plotted from the experiments.

**Figure 3:**
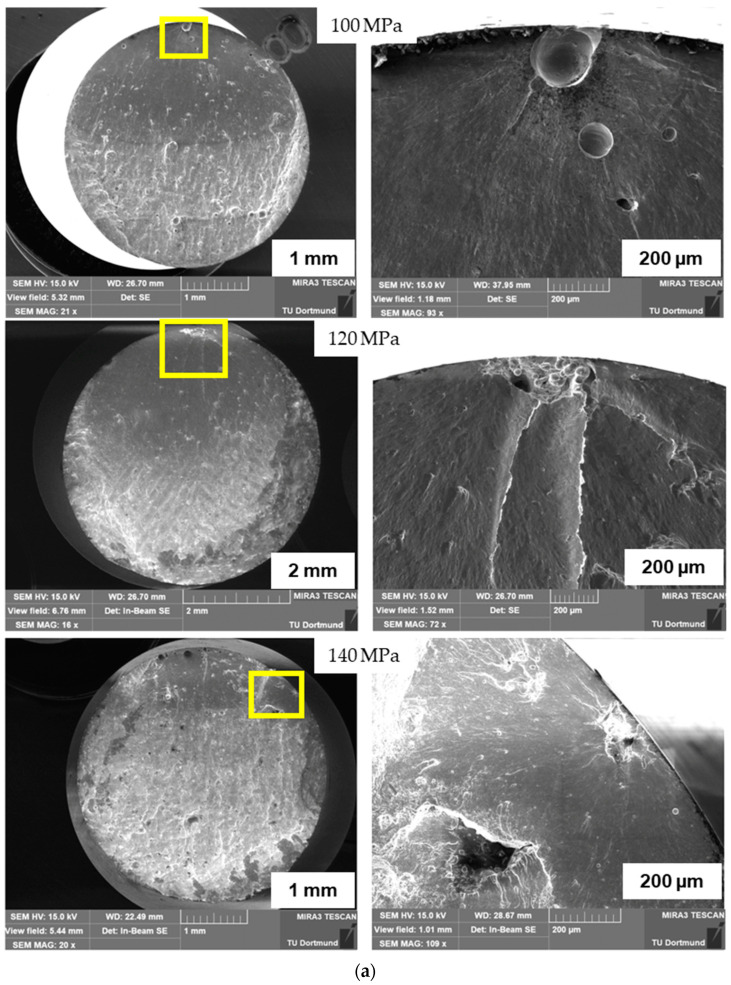

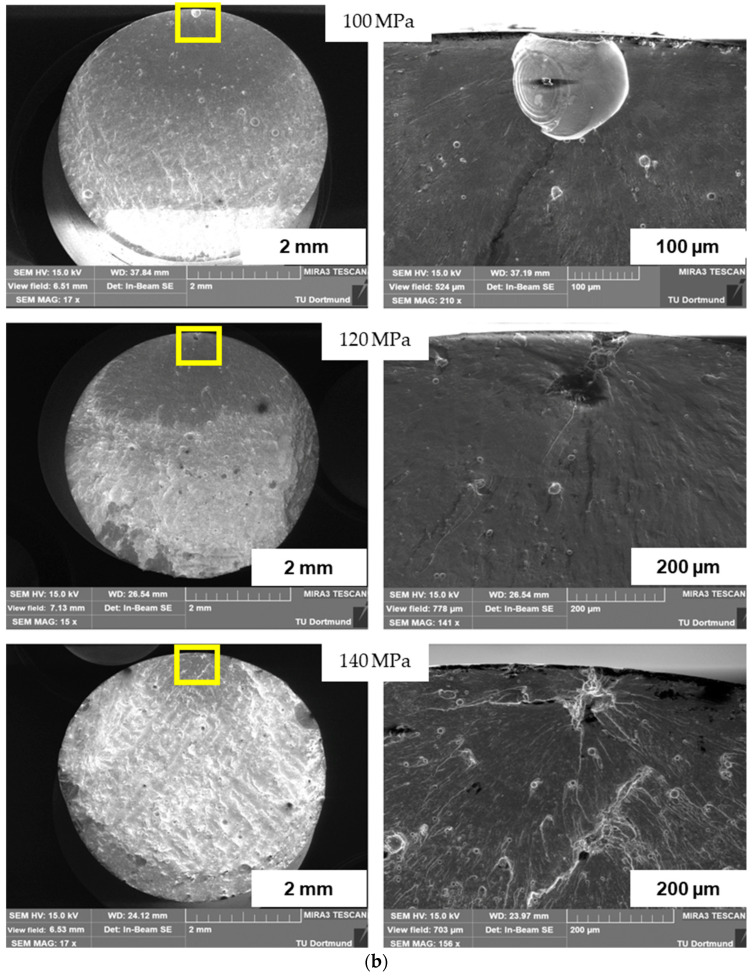
Fracture surfaces on SEM under 100 MPa, 120 MPa, and 140 MPa: (**a**) batch A; (**b**) batch B.

In this research, after the tensile test was performed, scanning electron microscopy (SEM) was carried out on the fracture surfaces of the tensile specimens. An overview (left) and a more in-depth look (right) are provided in Figure 3. It can be observed in the overview the fracture planes follow the direction of extensive porosity. Initiation from the subsurface flaw is visible in the detailed pictures on the right-hand side [52]. Figure 3 shows the scanning electron microscope results of fracture surfaces of tensile specimens of batch A (a) and batch B (b) under 100 MPa, 120 MPa, and 140 MPa loading.

### 3.2. Kitagawa–Takahashi Diagram Development

The original Kitagawa–Takahashi diagram (KT diagram) establishes the fatigue limits by combining the material’s intrinsic fatigue limit and the LEFM non-propagating crack condition [52,53]. The linear elastic fracture mechanics (LEFM) material’s characteristics for long cracks served as the basis for its development. The KT diagram was improved and adjusted for defects by Beretta and Romano [54]. However, the notch fatigue limit for porosity defects ought to serve as a lower limitation for the KT diagram. As a result, it is proposed in this research that the KT diagram for porosity defects contains three zones, as displayed here [52]. (a) Region I shows the applied stress being constant with the increase of defect size till the defect size reaches the intrinsic crack length. It is suitable when the size of the porosity is very small. (b) Region II establishes the non-propagating defect criteria under the LEFM condition of the SIF range equal to the threshold SIF range ($K=K_{th}$). Here, the slope ΔK value was determined from the finite element model for all the specimens. (c) Region III, which establishes the lower limit of the fatigue limit curve, is caused by the defect’s notch fatigue limit. This method was used in prior work to adjust the KT diagram for specimens with notches [55,56]. The lower bound was set using this formula [57] $\sigma_{lower\;bound}=\frac{\sigma_{fatigue\;limit}}{K_{t}}$. El-Haddad proposed in his pioneering research that the transition from Region I to Region 3 is rather more accurate when it follows this equation [57] $\Delta \sigma =\frac{\Delta K}{\sqrt{\pi \left(a+a_{0}\right)}}$; here, σ is the nominal stress range. To avoid inconsistency between the material fatigue limit and the defect and crack geometries, Equation (26) must be modified by the geometric factor Y [52,58].

(26) $$\Delta \sigma =\frac{\Delta K}{Y\sqrt{\pi \left(a+a_{0}\right)}}$$

From this above equation, the Kitagawa–Takahashi diagram and El-Haddad curve were developed for all four methods and the methods with correction factor hypothesis. Intrinsic defect length was calculated from the methods mentioned in theory, and defect length found from SEM was compared with the curve. The KT diagram of batches A and B under 120 MPa loading is shown here. CF stands for concentration factor.

**Figure 4:**
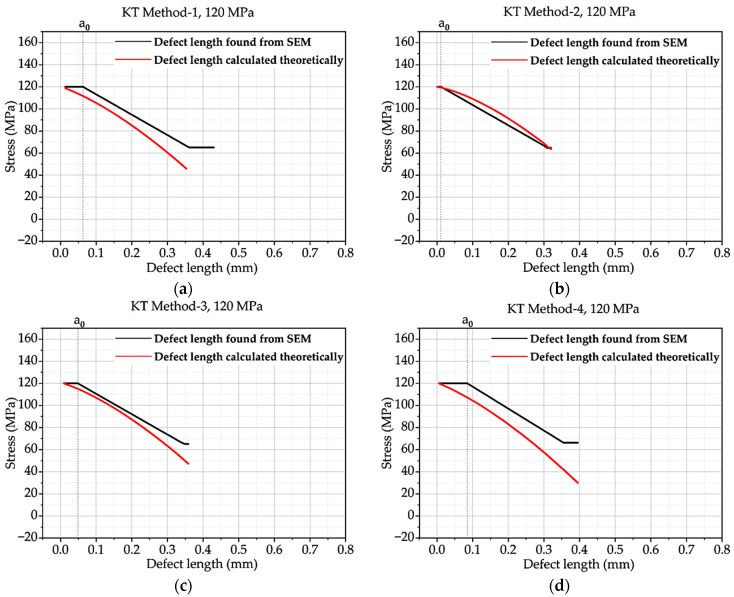
Kitagawa–Takahashi curve and El-Haddad curve for batch A under 120 MPa loading: (**a**) Method 1; (**b**) Method 2; (**c**) Method 3; (**d**) Method 4.

In Figure 4, the defect length discovered by SEM in Method-1 is much greater than the calculated defect length. The defect length determined by SEM in technique 2 is significantly smaller than the defect length predicted theoretically. In comparison to the outcomes for KT Method-1 or KT Method-2, the defect length discovered via SEM in Method-3 is closer to the defect length determined theoretically. The defect length determined by SEM in Method-4 matches the theoretically calculated defect length. 

The four charts demonstrate that the defect length discovered using SEM is not always identical to the defect length determined theoretically. The KT method employed affects the accuracy of the defect length determined from SEM.

**Figure 5:**
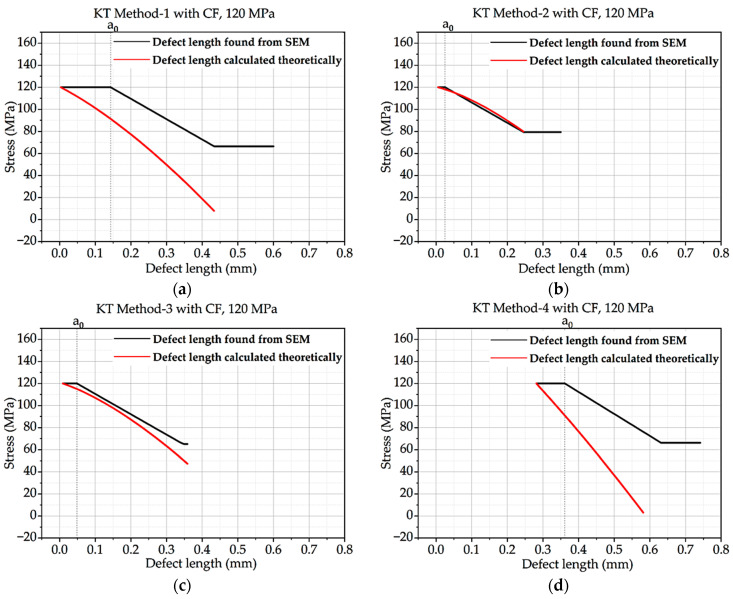
Kitagawa–Takahashi curve and El-Haddad curve for batch A with CF under 120 MPa loading: (**a**) Method 1; (**b**) Method 2; (**c**) Method 3; (**d**) Method 4.

The four diagrams in Figure 5 demonstrate the correlation between stress and defect length. With stress, the defect length increases. With regard to KT Method-1 and KT Method-2, the results for KT Method-3 and KT Method-4 are identical. The defect lengths by SEM vary significantly from the theoretically calculated defect lengths for KT Methods-1 and -2, but it is closer to the calculated defect lengths for KT Methods-3 and -4.

**Figure 6:**
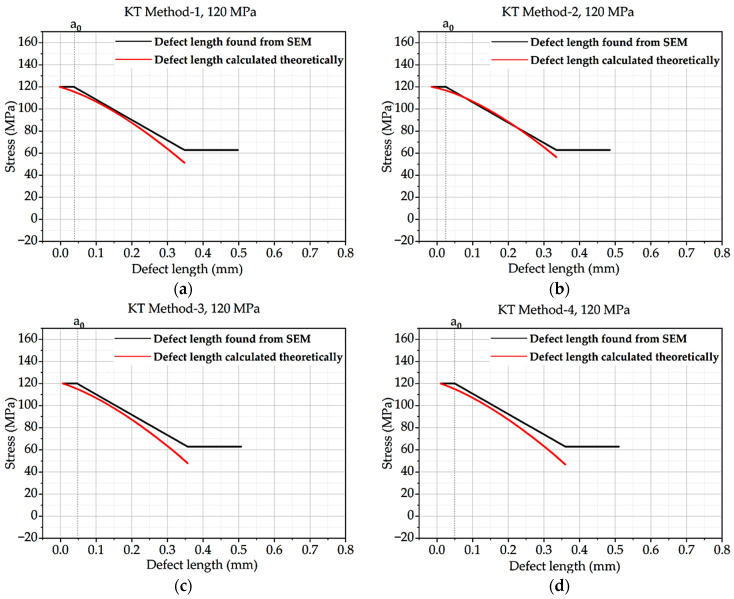
Kitagawa–Takahashi curve and El-Haddad curve for batch B under 120 MPa loading: (**a**) Method 1; (**b**) Method 2: (**c**) Method 3; (**d**) Method 4.

**Figure 7:**
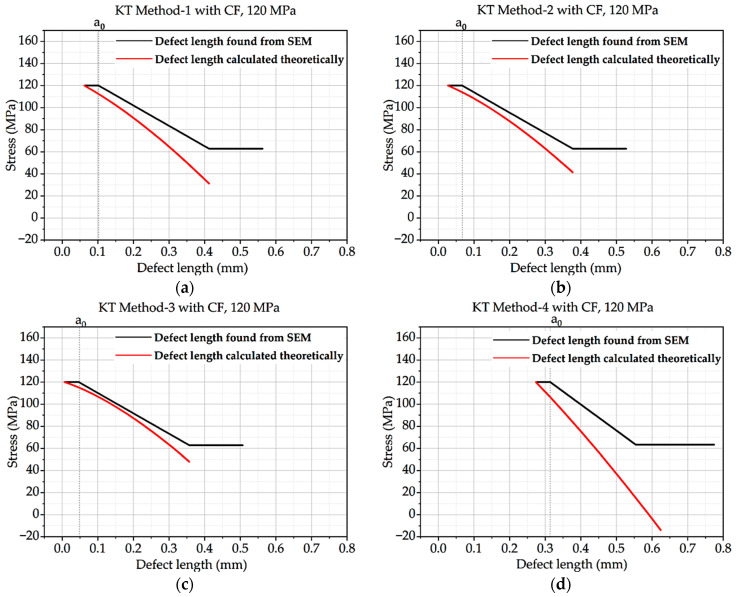
Kitagawa–Takahashi curve and El-Haddad curve for batch B with CF under 120 MPa loading: (**a**) Method 1; (**b**) Method 2: (**c**) Method 3; (**d**) Method 4.

The findings for KT Method-3 and KT Method-4 are closer to one another than the results for KT Method-1 and KT Method-2 in Figure 6 and Figure 7, respectively. In comparison to the other methods, the defect length discovered by SEM for KT Method-4 is closer to the defect length calculated theoretically.

### 3.3. Fatigue Life Calculation

To accurately predict the lifespan of fatigue, it is essential to collect a wide range of data points. Conducting thorough tensile tests allows us to quantify various mechanical properties, such as elastic modulus, stress-strain relationships, deformation energy, ultimate tensile strength, and yield strength for each studied specimen. Using scanning electron microscopy, we determined vital parameters such as the size and shape of the defect and grain. Additionally, finite element simulations were used to infer stress intensity factor values. This comprehensive research approach provides a solid foundation for accurate prognostications of fatigue life.

Within the theoretical framework, equations were applied to calculate key parameters such as intrinsic crack length, threshold stress intensity factors, and Paris’ law constants. Two distinct methodologies, Griffith’s theory, and the J-integral approach, were utilized to determine critical defect length and stress intensity factors. By incorporating the Paris equation, the complete cycle from force application to eventual failure was analyzed, leading to an accurate calculation of the fatigue lifespan. This research provides valuable insights into the mechanics of crack propagation and fatigue failure. Fatigue tests were conducted to determine the fatigue life of each specimen. For every batch, the highest and lowest values of fatigue life were taken, and an average was computed to enable comparison with the values derived from the present study.

**Figure 8:**
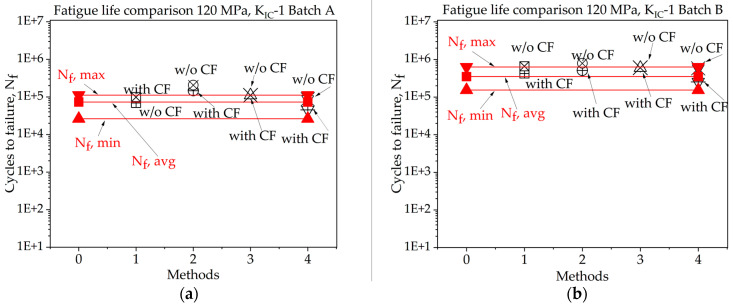
Fatigue life comparison under 120 MPa with *K_IC_*-1 of critical defect determination: (**a**) batch A; (**b**) batch B.

**Figure 9:**
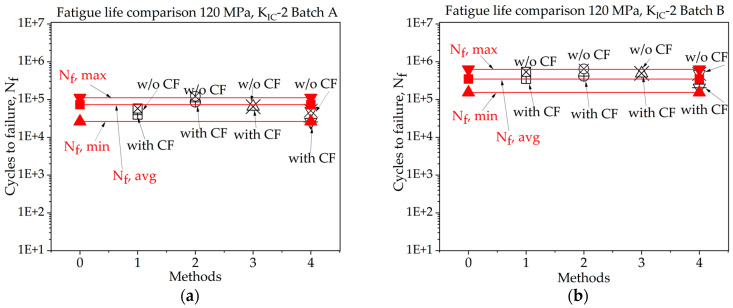
Fatigue life comparison under 120 MPa with *K_IC_*-2 of critical defect determination: (**a**) batch A; (**b**) batch B.

**Figure 10:**
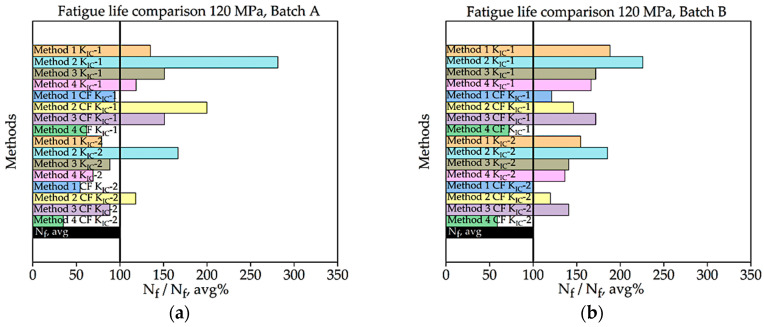
Percentage ratio of theoretical $N_{f}$ and experimental $N_{f,avg}$ under 120 MPa loading: (**a**) batch A; (**b**) batch B.

**Figure 11:**
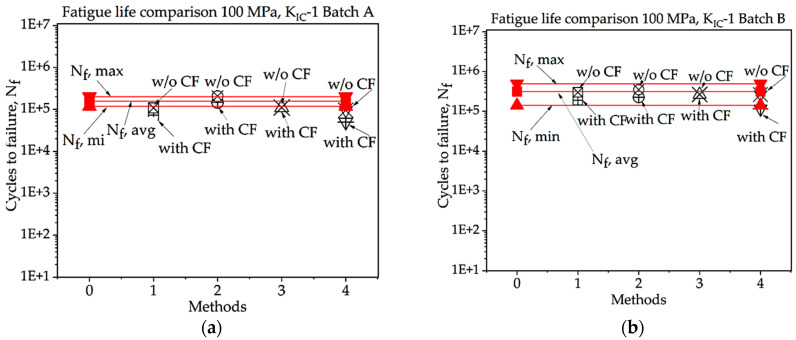
Fatigue life comparison under 100 MPa with *K_IC_*-1 of critical defect determination: (**a**) batch A; (**b**) batch B.

**Figure 12:**
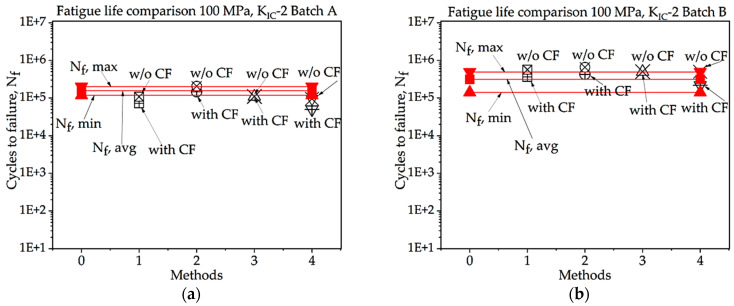
Fatigue life comparison under 100 MPa with *K_IC_*-2 of critical defect determination: (**a**) batch A, (**b**) batch B.

**Figure 13:**
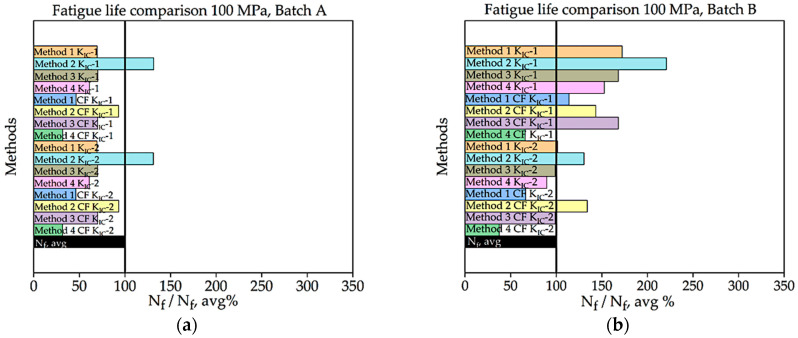
Percentage ratio of theoretical $N_{f}$ and experimental $N_{f,avg}$ under 100 MPa loading: (**a**) batch A; (**b**) batch B.

**Figure 14:**
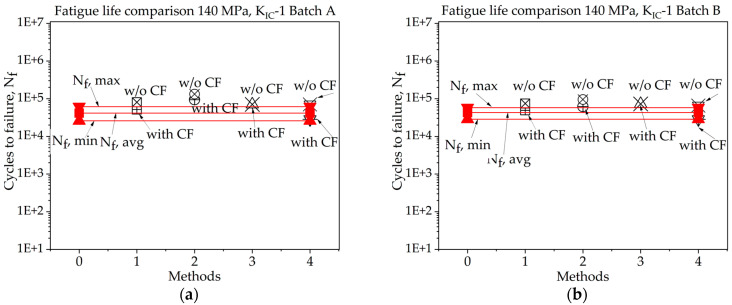
Fatigue life comparison under 140 MPa with *K_IC_*-1 of critical defect determination: (**a**) batch A; (**b**) batch B.

**Figure 15:**
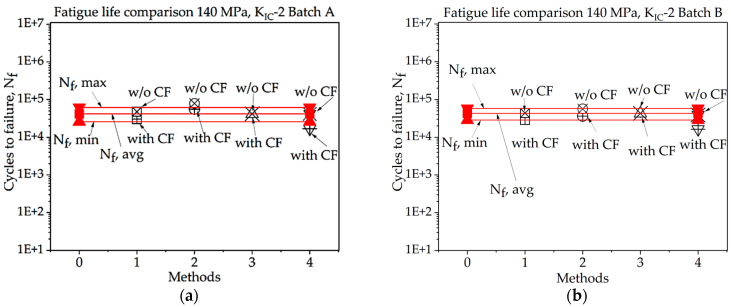
Fatigue life comparison under 140 MPa with *K_IC_*-2 method of critical defect determination: (**a**) batch A; (**b**) batch B.

**Figure 16:**
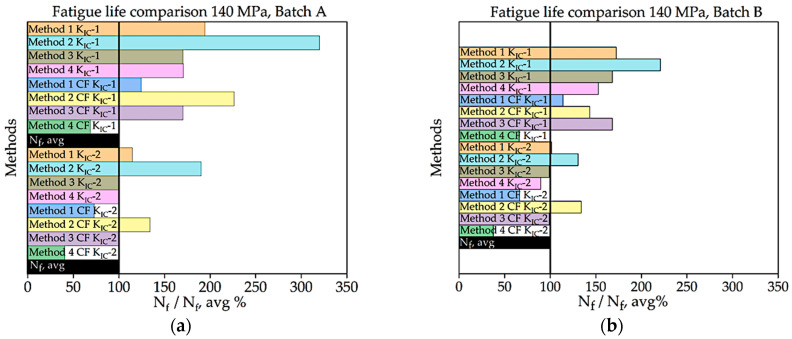
Percentage ratio of theoretical $N_{f}$ and experimental $N_{f,avg}$ under 140 MPa loading: (**a**) batch A; (**b**) batch B.

In Figure 8, Figure 9, Figure 10, Figure 11, Figure 12, Figure 13, Figure 14, Figure 15 and Figure 16 it is revealed that batch A specimens subjected to 120 MPa loading, determined using $K_{IC}$, Method 1 with the correction factor, yields the highest accuracy for fatigue life, at 94.20%. While using this method, the precision of the results was overall suboptimal. When employing $K_{IC}$, Method 3 attains the highest accuracy at 88.60%. The precision of the results found in all four methods is higher in this case than in the other one. Though 1st method with CF while using $K_{IC}$ provides the best result for fatigue life, using $K_{IC}$ overall precision in all methods was found to be superior. For batch B specimen under 120 MPa loading, using $K_{IC}$*,* Method 2 with the correction factor achieved the best results, with a 21.36% error. While using $K_{IC}$, Method 1 with CF procures the highest accuracy for fatigue life, with an error rate of only 0.29%. In this case, the precision across all four methods is superior compared to $K_{IC}$. Here, Method 1 with CF is proved to be the most accurate to determine fatigue life.

In Figure 8, Figure 9, Figure 10, Figure 11, Figure 12, Figure 13, Figure 14, Figure 15 and Figure 16, for batch A specimens under 100 MPa loading, using $K_{IC}$, Method 2 with CF gives the result with the highest accuracy, which is 93%. While using $K_{IC}$, Method 2 with CF garners the most accurate result, at 93.33%. In both methods of $K_{IC}$ calculation, the results are quite similar. Here, Method 2 with CF provides the best result for fatigue life.

For batch B specimens, it was found that using $K_{IC}$, Method 1 without CF, generates the most accurate result, at 95.70%. Method 2, 3, and 4 without correction factor also produce results with only 10–15% error. Here, the precision of the results found in all four methods is higher than the other. While considering $K_{IC}$, Method 1 with the CF gives the best value for fatigue life, with an accuracy of 115.90%. The precision of the results found here was overall poor, and several values crossed the experimental threshold range of fatigue life.

For batch A specimen under 140 MPa loading, while considering $K_{IC}$, Method 1 with CF procured the highest accuracy, at 124.55%. In this case, the precision of the results was poor overall. While using $K_{IC}$, methods 3 and 4 without CF achieve the best results, with nearly 100% accuracy. In this scenario, the precision across all four methods is higher, and only one fatigue life value exceeds the experimentally determined threshold.

For batch B specimens under 140 MPa loading, using the 1st method of $K_{IC}$ calculation, Method 1 with CF gives the highest accuracy, which is 114%. The precision of the results found in all four methods is lower in this case than the other one. While considering the 2nd method to determine $K_{IC}$, Method 1 and Method 3 without the correction factor give the best value for fatigue life, with an accuracy of 101.50% and 99% and an error rate of 1.5% and 1.0%, respectively. While using this method, only one of the determined fatigue lives crossed the experimental threshold range of fatigue life, and others are highly accurate.

## 4. Conclusions

In this study, for each specimen, a total of eight values of fatigue life were determined using methods mentioned in theory. After determining the fatigue life from these methods, they were compared with the fatigue life results found from the fatigue life experiment under 100 MPa, 120 MPa, and 140 MPa cyclic loading conditions. Here, different methods stood out for different loading conditions. The best methods for each condition are listed below in Table 1.

Here, it can be seen that both batch A and batch B respond similarly to the methods depending on the loading conditions. For 120 MPa loading, the 1st method with correction factor using the first method to determine $K_{IC}$ gives the most reliable result for both batches A and B. For 100 MPa, the 2nd method with CF using both methods of $K_{IC}$ calculation for batch A and the 1st method with CF using the first method of $K_{IC}$ calculation for batch B gives the closest result to the experimental value. For 140 MPa, the 3rd method using the second method of $K_{IC}$ calculation gives the best resemblance to the experimental results. Here, Method 1 with correction factor (in a few cases without CF) has provided the best average results among all the methods for different conditions. Method 2 was proven very reliable for low loading conditions, where as much as the load increases, it provides lower accuracy. In Table 2, Methods 1, 3, and 4 were more reliable for higher loading conditions. Here, it can be seen that, due to different loading conditions, internal defect length also becomes different, and to predict fatigue life, different methods have to be used due to the difference in these loading conditions. Overall, this study provides several reliable methods to determine fatigue life and shows the effect of internal defects on fatigue characteristics. The findings of this study have important implications for the design and manufacturing of SLMed AlSi10Mg components. Different loading conditions were considered to study the effects of defects on fatigue life. The internal defects reduce the fatigue life significantly at different loading conditions, and by understanding such inverse effects of internal defects on fatigue life, the engineers can design components that are more resistant to fatigue failure. Additionally, manufacturers can also optimize the SLM process to reduce the occurrence of internal defects and improve the fatigue performance of their products.

## Figures and Tables

**Table 1:** Best methods to determine fatigue life under 100 MPa, 120 MPa, and 140 MPa loading for batch A and batch B.

Batch	Loading Condition	Best Method	Error Percentage
A	120	1st with CF using *K_IC_*-1	5.8%
A	100	2nd with CF using *K_IC_*-1 and 2	6.66%
A	140	3rd and 4th without CF using *K_IC_*-2	0.5%
B	120	1st with CF using *K_IC_*-1	0.29%
B	100	1st without CF using *K_IC_*-1	4.3%
B	140	1st and 3rd without CF using *K_IC_*-2	1%

**Table 2:** The percentage ratio of theoretical $N_{f}$ and experimental $N_{f,avg}$ under 100 MPa loading.

Stress			100 MPa				120 MPa				140 MPa		
*K_IC_* Method		*K_IC_*-1		*K_IC_*-2		*K_IC_*-1		*K_IC_*-2		*K_IC_*-1		*K_IC_*-2	
batch A		*N_f_/N_f,avg_%*	Error%	*N_f_/N_f,avg_%*	Error%	*N_f_/N_f,avg_%*	Error%	*N_f_/N_f,avg_%*	Error%	*N_f_/N_f,avg_%*	Error%	*N_f_/N_f,avg_%*	Error%
	Method 1	135%	−35%	79%	21%	70%	30%	69%	31%	194%	−94%	115%	−15%
	Method 2	282%	−182%	167%	−67%	132%	−32%	131%	−31%	320%	−220%	190%	−90%
	Method 3	151%	−51%	89%	11%	70%	30%	70%	30%	170%	−70%	100%	0%
	Method 4	119%	−19%	69%	31%	61%	39%	61%	39%	171%	−71%	101%	−1%
	Method 1 CF	94%	6%	54%	46%	47%	53%	46%	54%	125%	−25%	73%	27%
	Method 2 CF	200%	−100%	118%	−18%	93%	7%	93%	7%	226%	−126%	134%	−34%
	Method 4 CF	62%	38%	35%	65%	32%	68%	32%	68%	69%	31%	41%	59%
batch B	Method 1	188%	−88%	155%	−55%	96%	4%	181%	−81%	173%	−73%	102%	−2%
	Method 2	226%	−126%	185%	−85%	110%	−10%	209%	−109%	221%	−121%	131%	−31%
	Method 3	172%	−72%	141%	−41%	84%	16%	159%	−59%	168%	−68%	99%	1%
	Method 4	167%	−67%	136%	−36%	84%	16%	160%	−60%	153%	−53%	90%	10%
	Method 1 CF	121%	−21%	100%	0%	61%	39%	116%	−16%	114%	−14%	66%	34%
	Method 2 CF	146%	−46%	120%	−20%	72%	28%	136%	−36%	143%	−43%	84%	16%
	Method 4 CF	72%	28%	59%	41%	37%	63%	70%	30%	66%	34%	38%	62%

## Data Availability

Not applicable.
